# Evolution of *Streptococcus pyogenes* has maximized the efficiency of the Sortase A cleavage motif for cell wall transpeptidation

**DOI:** 10.1016/j.jbc.2022.101940

**Published:** 2022-04-14

**Authors:** Bradley M. Readnour, Yetunde A. Ayinuola, Brady T. Russo, Zhong Liang, Shaun W. Lee, Victoria A. Ploplis, Vincent A. Fischetti, Francis J. Castellino

**Affiliations:** 1 W. M. Keck Center for Transgene Research, University of Notre Dame, Notre Dame, Indiana, USA; 2 Department of Chemistry and Biochemistry, University of Notre Dame, Notre Dame, Indiana, USA; 3 Department of Biological Sciences, University of Notre Dame, Notre Dame, Indiana, USA; 4 Laboratory of Bacterial Pathogenesis and Immunology, Rockefeller University, New York, New York, USA

**Keywords:** bacteria, bacterial pathogenesis, cell surface, cell wall, ligand-binding protein, c2ll membrane enzymes, subcellular fractionation, mutagenesis, plasminogen, *Streptococcus pyogenes*, BSA, bovine serum albumin, CM, cytoplasmic membrane, CW, cell wall, DCO, double crossover, EM, extracellular membrane, GAS, Group A Streptococcus pyogenes, gDNA, genomic DNA, hPg, human plasminogen, HVD, hypervariable domain, Mprt, M-protein, SCO, single crossover, Sec, secretory, SrtA, sortase A, TMD, transmembrane region

## Abstract

Trafficking of M-protein (Mprt) from the cytosol of Group A *Streptococcus pyogenes* (GAS) occurs *via* Sec translocase membrane channels that associate with Sortase A (SrtA), an enzyme that catalyzes cleavage of Mprt at the proximal C-terminal [-LPST^355∗^GEAA-] motif and subsequent transpeptidation of the Mprt-containing product to the cell wall (CW). These steps facilitate stable exposure of the N-terminus of Mprt to the extracellular milieu where it interacts with ligands. Previously, we found that inactivation of SrtA in GAS cells eliminated Mprt CW transpeptidation but effected little reduction in its cell surface exposure, indicating that the C-terminus of Mprt retained in the cytoplasmic membrane (CM) extends its N-terminus to the cell surface. Herein, we assessed the effects of mutating the Thr^355^ residue in the WT SrtA consensus sequence (LPST^355∗^GEAA-) in a specific Mprt, PAM. *In vitro*, we found that synthetic peptides with mutations (LPSX^355^GEAA) in the SrtA cleavage site displayed slower cleavage activities with rSrtA than the WT peptide. Aromatic residues at X had the lowest activities. Nonetheless, PAM/[Y^355^G] still transpeptidated the CW *in vivo*. However, when using isolated CMs from *srtA*-inactivated GAS cells, rapid cleavage of PAM/[LPSY^355^GEAA] occurred at E^357∗^ but transpeptidation did not take place. These results show that another CM-resident enzyme nonproductively cleaved PAM/[LPSYGE^357∗^AA]. However, SrtA associated with the translocon channel *in vivo* cleaved and transpeptidated PAM/[LPSX^355^^∗^GEAA] variants. These CM features allow diverse cleavage site variants to covalently attach to the CW despite the presence of other potent nonproductive CM proteases.

The surface of Gram-positive Group A *Streptococcus pyogenes* (GAS) is decorated with a variety of proteins that function in aiding the survival and virulence of these microorganisms. Unlike Gram-negative bacteria which use their outer membranes to stabilize proteins on the cell surface, Gram-positive bacteria must employ export channels, cytoplasmic membranes (CMs), and relatively thick cell wall (CW) peptidoglycans to anchor their surface proteins. Surface protein exposure in these cells occurs by at least three mechanisms (1), specifically: (a) moonlighting proteins bound *via* hydrophobic/charge interactions to the CW/outer capsule/teichoic acids/group carbohydrate, for example, streptococcal enolase (2, 3); (b) proteins inserted in the CM that can extend to the cell surface, for example, lipoproteins (4); and (c) proteins covalently linked by posttranslational processes to the CW, for example, M- and M-like proteins (5). All protein epitopes exposed on the cell surfaces of Gram-positive bacteria are stabilized by one or more of these processes.

Prokaryotic proteins that are ribosomally generated in the cytoplasm must be secreted or translocated into various cell compartments. This trafficking of different classes of proteins from the cytosol possesses some unique features (6). Information contained within the signal peptide and other regions of the protein sequence directs transport of proteins to their cellular compartments through specific CM channels (7, 8). Cytosolic proteins targeted for extracellular secretion are synthesized with an N-terminal cleavable signal peptide and are transported in their unfolded states through general secretory (Sec) translocon channels, which are assembled in microdomains in the CM from partner proteins. On the other hand, some surface displayed proteins, broadly classified as moonlighting proteins (9), for example, enolase, which functions in the cytoplasm in glycolysis but has other properties on the cell surface, contain neither a signal sequence nor other known trafficking information but nonetheless leave the cytosol and are fully or partly exposed on the cell surface. In Gram-positive GAS, these proteins are likely stabilized by hydrophobic/charge interactions with the CW/outer capsule components or can even extend from the CM to the cell surface.

Another group of surface displayed proteins also contains cleavable N-terminal signal sequences which terminate in a lipobox (LAAC), for example, lipoproteins (7). These lipoproteins are enzymatically diacylated (with a diacyl glyceryl moiety) at the side-chain SH of the latent Cys^1^-residue and then are stably inserted into the outer leaflet of the CM in Gram-positive bacteria, where the signal sequence is cleaved, leaving the N-terminal Cys embedded in the CM *via* its acyl groups. This allows extension of the C-terminal regions of some of these proteins to the cell surface.

A final group of proteins, for example, M-proteins (Mprts), contain cleavable N-terminal signal sequences within which is a YSIRK amino acid sequence motif (10) that directs their trafficking to the multiprotein Sec translocon and also encode a transmembrane region (TMD) near the C-terminus. Using this latter example, Mprts are delayed in the CM at the translocon by their TMDs where they are further processed by CM-embedded Sortase (Srt)-type transpeptidases, enzymes that are also located at the Sec channel (11). Housekeeping SrtA, universally present in Gram-positive bacteria, recognizes a pentapeptide CW sorting signal, for example, LPXTG, followed by a hydrophobic region of ∼21 residues and a short positively charged cytoplasmic tail (12). SrtA is a Cys-proteinase that functions by forming an acyl-enzyme complex *via* nucleophilic attack of the active site Cys at the T^↓^G of the LPXTG signal motif. This step yields a thioacyl intermediate complex, which then transfers the product to Lipid II. The Lipid II–linked intermediate then becomes a substrate for the growing peptidoglycan CW (13), which results in the covalent attachment of the processed protein to the CW *via* the transpeptidase activity of SrtA (14, 15).

Srts are very important to the composition and consequent properties of the CW and the cell surface and have also been used artificially to engineer the surface of cells (16). Thus, a deeper understanding of the properties of these enzymes is of great importance.

## Results

**Figure 1:**
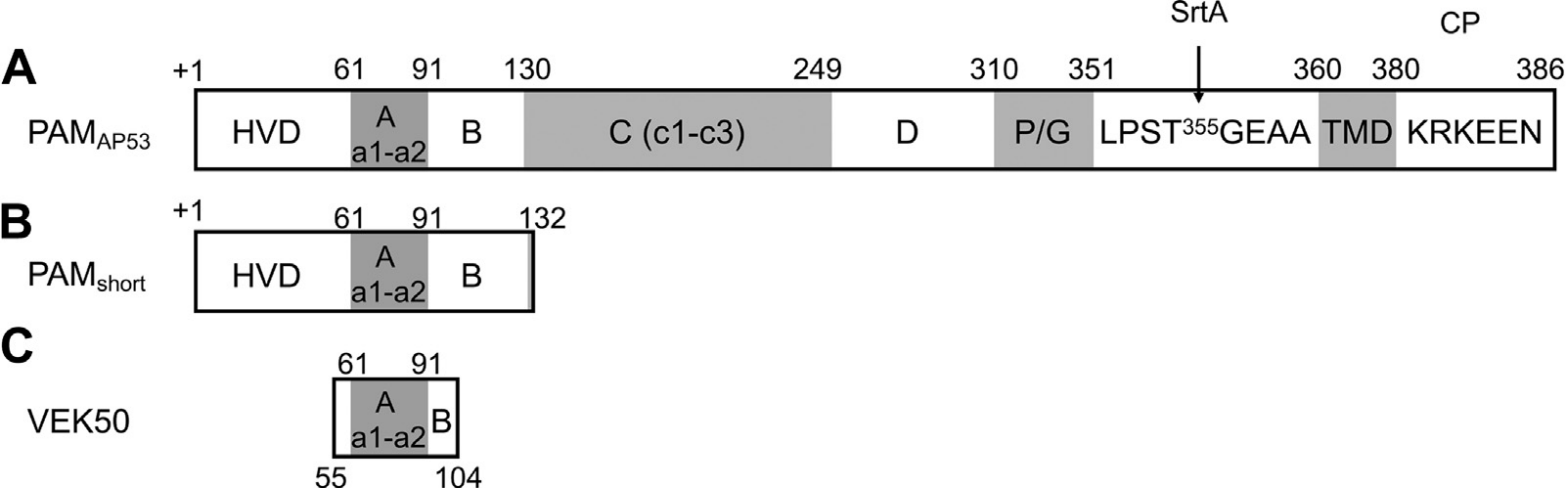
**Domain structure of PAM**_**AP53**_**.***A*, the 427 amino acid-residue PAM protein sequentially consists of a 41-residue signal polypeptide, immediately downstream of which is a hypervariable domain (HVD), followed by an A-domain with two hPg binding a-repeats, a B-domain, a C-domain with three c-repeats, a D domain, a Pro/Gly region, a sortase A (Srt A) recognition motif, a transmembrane domain (TMD) and, lastly, a short C-terminal cytoplasmic insertion sequence (CP). Recombinant PAM spans residues 1 to 348. T^355^, the mutagenesis site in this study, is highlighted (18). *B*, PAM_short_ is a recombinant construct consisting of residues 1 to 132 and consists of the full HVD, the full hPg binding A-domain, and the full B-domain, with 3-residues of the C-domain. *C*, VEK50 is a form of PAM consisting of residues 55 to 104 and contains the C-terminus of the HVD, the full A-domain, and the N-terminus of the B-domain. hPg, human plasminogen.

For ease in reading this article, the domain structures of the Pattern D GAS-AP53 Mprt, PAM_AP53_, along with important recombinant fragments of PAM_AP53_, are diagrammed in Figure 1. PAM is the only Mprt present in Pattern D GAS cells and is used for *emm* serotyping of the GAS (*emm53* in this case).

### Cleavage of synthetic peptides by SrtA

From the mining of our annotated genomic sequence of GAS-AP53 (GenBank: CP013672.1) (17), we find that the only Srts present in GAS-AP53 cells are SrtA (GenBank: AMY97447.1) and SrtB (GenBank: AMY96673.1). The specificities of these Srts are very different and only SrtA strictly applies to this study. SrtB was used as a negative control for SrtA in some experiments.

**Figure 2:**
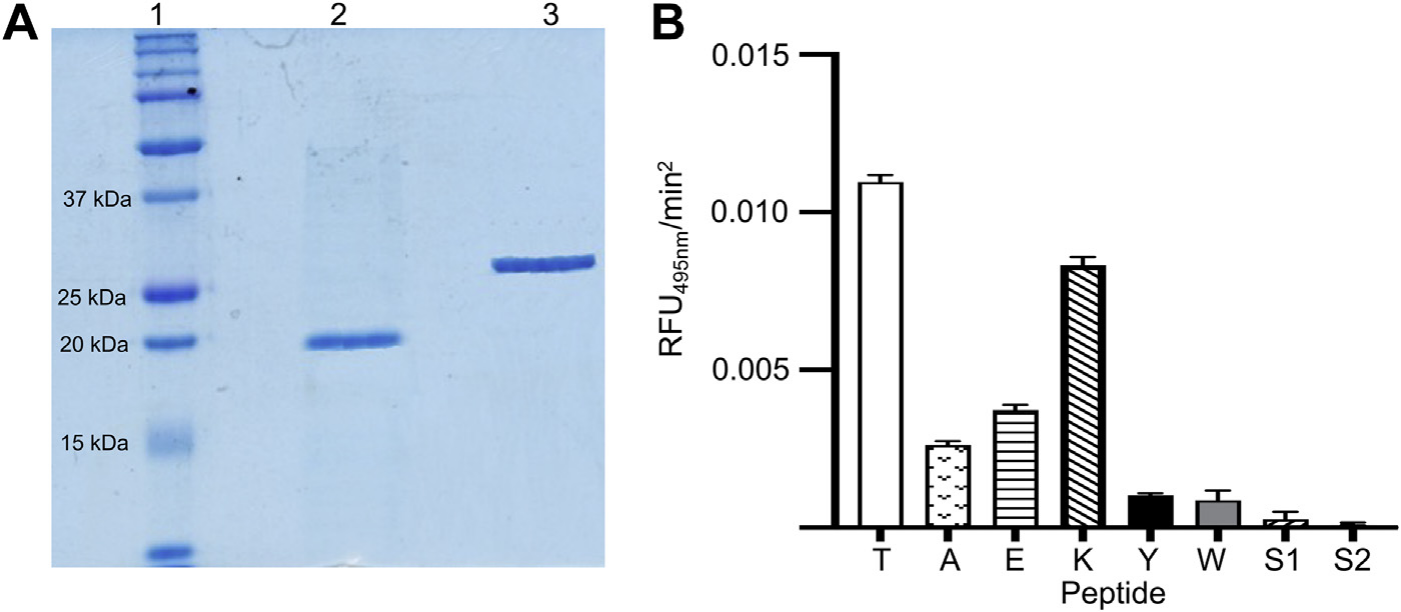
**Purification and activity of recombinant sortases**. *A*, SDS-PAGE gels of purified recombinant (r) [V^82^]SrtA and [H^37^]SrtB. Lane 1, molecular weight markers. Lane 2, r[V^82^]SrtA. Lane 3, r[H^37^]SrtB. *B*, cleavage of fluorescent peptides catalyzed by r[V^82^]SrtA. Dabcyl-LPSXGEAA-Edans peptides (X = T, A, E, K, Y, W) and two scrambled peptides, *viz*., S1, Dabcyl-YLGPSEAA-Edans and S2, Dabcyl-TLPGSEAA-Edans were incubated with r[V^82^]SrtA and the relative fluorescence are measured. The peptides were excited at 350 nm, and emission was measured at 495 nm. None of the peptides showed cleavage when catalyzed by r[H^37^]SrtB. N = 3 for each condition.

To purify both Srts, recombinant (His)_6_-tagged [V^82^]SrtA and [H^37^]SrtB were expressed in *Escherichia coli* cells, purified by affinity chromatography on columns containing immobilized Ni^2+^, and eluted with imidazole. The final proteins were of high purity and appear at the correct molecular weights (Fig. 2*A*). These purified enzymes were then employed to examine the cleavage by FRET of the fluorescence-tagged substrates, Dabcyl-LPSXGEAA-Edans, where X = T, A, E, K, W, or Y. The results of the assay are shown in Figure 2*B*. While all substrates are cleaved at different rates by SrtA, no linear rate of cleavage was faster than that of the native LPSTGEAA and the lowest initial rates of cleavage catalyzed by SrtA were found for the peptides with aromatic residues substituted at sequence position-4, *viz*., LPSYGEAA and LPSWGEAA.

### Subcellular distribution of PAM in isogenic AP53 cells

**Figure 3:**
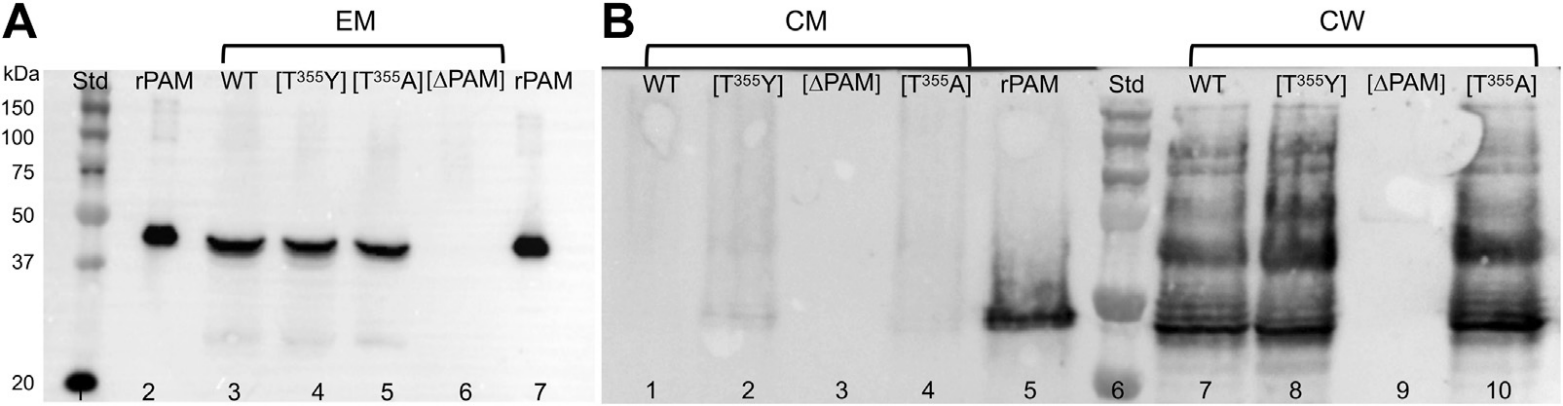
**PAM distributions in fractionated mid-log phase cells.***A*, Western blot analysis of the extracellular medium (EM) with anti-PAM_short_. The extracellular medium (EM) samples were concentrated 40× prior to analysis. Lane 1, molecular weight ladder. Lane 2, rPAM; Lanes 3 to 6 contained EMs (40× concentrated) from cells of the following: lane 3, WT-AP53; lane 4, WT-AP53/*pam[T*^*355*^*Y]*; lane 5, AP53/*pam[T*^*355*^*A]*; and lane 6, AP53/*Δpam*. Lane 7 is rPAM. *B*, AP53 cells were washed and digested with Ply-C. Cell wall (CW) and cytoplasmic membrane (CM) fractions were analyzed by Western blotting with anti-PAM_short_. Lanes 1 to 4 represent the CM fractions from the following: lane 1, WT-AP53; lane 2, AP53/*pam[T*^*355*^*Y]*; lane 3, AP53/*Δpam*; and lane 4, AP53/*pam[T*^*355*^*A]* cells. Lane 5 is rPAM and lane 6 is a standard molecular weight ladder. Lanes 7 to 10 are CW fractions from the following: lane 7, WT-AP53; lane 8, AP53/*pam[T*^*355*^*Y]*; lane 9, AP53/*Δpam*; and lane 10, AP53/*pam[T*^*355*^*A]* cells.

Since WT-PAM (GenBank: AMY98239.1) was selectively retained in the CM in cells in which SrtA was inactivated (18), we designed experiments to determine the cellular distribution of AP53/PAM[T^355^Y] and AP53/PAM[T^355^A], compared to WT-PAM, in cell lines with a targeted substitution of these variants in place of WT-PAM. We began by evaluating whether these SrtA-resistant PAMs were processed and secreted in the extracellular medium (EM) at mid-log phase cell growth (Fig. 3*A*). Western blot assays with polyclonal rabbit-anti-PAM and rabbit-anti PAM_short_ did not reveal meaningful differences in cell surface expression between the SrtA cleavage product of WT-PAM (lane 3, molecular weight ∼40.6 kDa) and the PAM variants (lanes 4 and 5, molecular weights ∼40.6 kDa) of these different cell lines. Moreover, since the Western blot analyses were performed using anti-PAM_short_, the data show that the cell surface PAM variants contain their N-termini, which is primarily the A-domain of PAM. The molecular weights of the PAM variants are the same as that of WT-PAM and all are nearly identical to recombinant PAM (lanes 2 and 7, molecular weight ∼40.9 kDa), which has extra residues for purification purposes, with no band appearing for the AP53/*Δpam* cells (lane 6), as anticipated. These results demonstrate that PAM[T^355^Y] and PAM[T^355^A] have been cleaved by an enzyme(s) that recognizes the SrtA cleavage sequences, LPSAGEAA and LPSYGEAA.

We next examined the distribution of PAM in mid-log phase subcellular fractions isolated from WT-AP53, AP53/*pam[T*^*355*^*A]*, and AP53/*pam[T*^*355*^*Y*] cells. The results of the experiments are shown in Figure 3*B*. There is very little PAM in the CM fractions obtained from WT-AP53 (lane 1), AP53/*pam[T*^*355*^*Y]* (lane 2), and AP53/*pam[T*^*355*^*A]* cells (lane 4) and, as expected, PAM was not present in AP53/*Δpam* cells (lane 3). However, PAM was observable in the CW fractions of WT-cells (lane 7), as well as in both isogenic mutant strains (lanes 8 and 10), but not in AP53/*Δpam* cells (lane 9). Thus, the PAM variants were cleaved to a similar extent as WT-PAM, despite the reduced level of activity that the synthetic peptides showed against rSrtA *in vitro*. Further, these variants were covalently attached to the CW, as indicated by the higher molecular weight PAM-positive bands that contained partially digested CW peptidoglycan, similar to WT cells. This demonstrates that the PAM variants are processed through SrtA. A reasonable explanation of these results is that SrtA, *in vitro*, has a lower level of activity toward its cleavage site in synthetic peptides than SrtA present in the translocon channel (19). While the SrtA cleavage site peptide variants exhibit reduced activity toward SrtA *in vitro*, the activity is sufficient in cells to both cleave and transfer these variants to the CW when these sequences are present *in vivo* in PAM.

### Expression of WT-PAM and its Srt motif variants on the cell surface of isogenic AP53 strains

**Figure 4:**
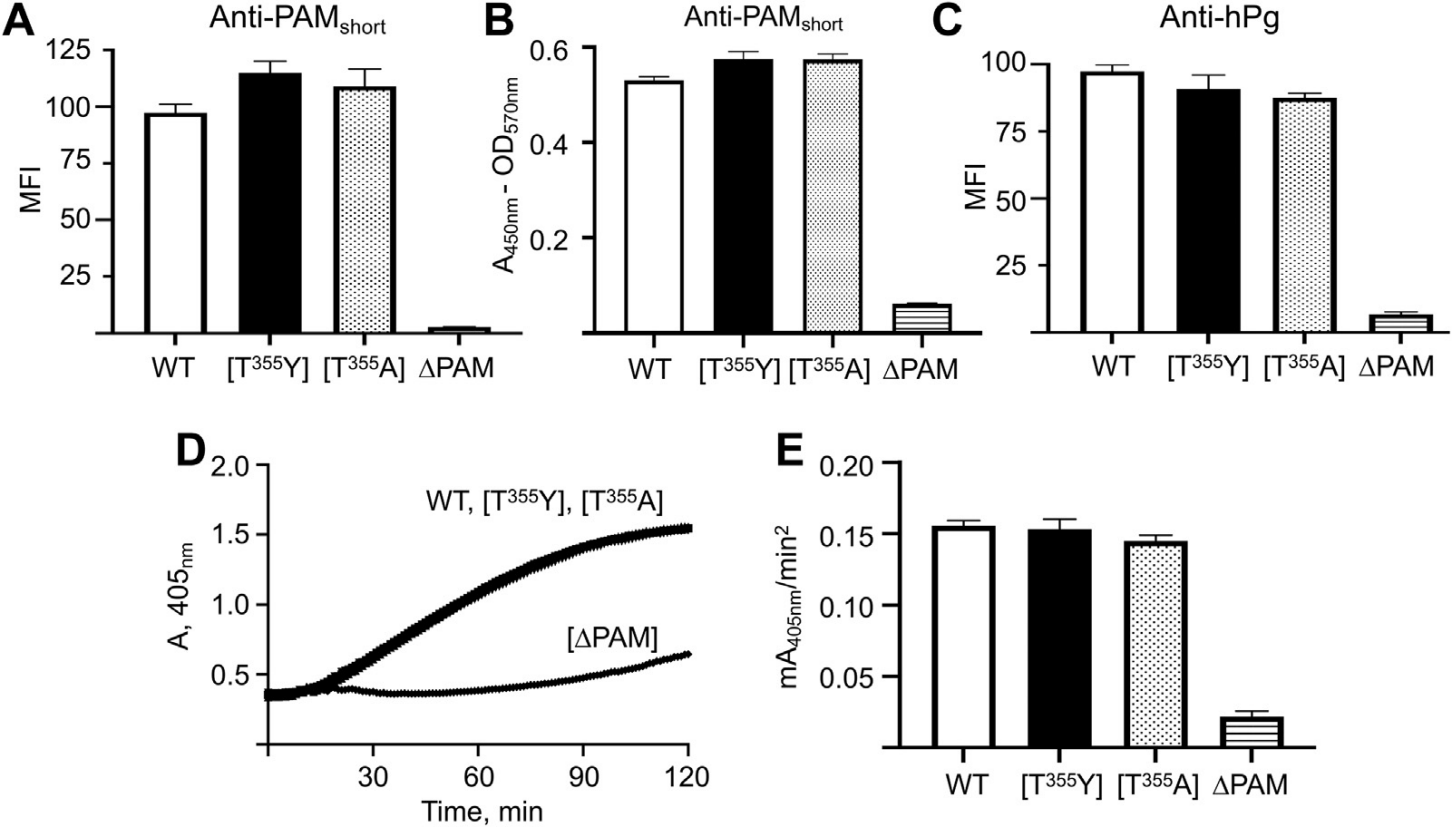
**Binding and activation of hPg by variant PAMs.***A*, FCA of rabbit-anti-PAM binding to whole AP53 cells. WT-AP53, AP53/*pam[T*^*355*^*Y]*, AP53/*pam[T*^*355*^*A]*, and AP53/*Δpam* mid-log-phase cells were incubated with rabbit-anti-PAM_short_ followed by Alex Fluor 488-chicken-anti-rabbit IgG. The median fluorescence intensities (MFI) determined by FCA are shown in a *bar graph* of the relative levels of PAM on each of the different cell lines. *B*, *ELISA* of rabbit-anti PAM_short_ binding to whole AP53 cells. Bar graph of the data is from the incubation with rabbit-anti PAM_short_ as in (*A*), followed by goat-anti-rabbit IgG-HRP. Equal numbers of cells were added to wells of a 96-well plate, and the HRP substrates, H_2_O_2_, and TMB were added to each well. The reaction was terminated with H_2_SO_4_. The A_570nm_ due to light scatter was subtracted from A_405nm_. *C*, *hPg binding to isogenic AP53 strains*. Mid-log phase AP53 cells were treated with hPg and incubated with monoclonal mouse-anti hPg IgG. The bound hPg was detected by incubation with Alex Fluor 488-donkey-anti-mouse IgG, analyzed as in (A), and presented as a *bar graph*. *D*, *hPg activation by SK2b*. The isogenic AP53 cells were placed in individual wells of a protein nonbinding 96-well microtiter plate, after which hPg was added, followed by S2251/SK2b. The hPm-catalyzed release of p-nitroaniline from S2251 was continuously monitored at 405 nm for 120 min. *E*, *hPg activation by SK2b*. The initial rates of activation calculated from the curves in D are represented as bar graphs. At least three different biological replicates were used for each strain. The buffer was 10 mM Na-Hepes/150 mM NaCl, pH 7.4, at room temperature. N ≥ 3 for each experiment. FCA, flow cytometric analysis; hPg, human plasminogen;; HRP, horseradish peroxidaseTMB, tetramethylbenzidine.

To confirm the presence and integrity of the PAM variants on the AP53 cell surface, we performed whole cell immunoassays using anti-PAM_short_, as well as flow cytometric analysis of human plasminogen (hPg) binding to the cell lines. The data of Figure 4 show that equivalent amounts of each PAM are present on AP53 cells with their N-termini largely intact, as revealed by their reactivities toward rabbit-anti-PAM_short_, when whole cells are analyzed by flow cytometric analysis (Fig. 4*A*) and by ELISA (Fig. 4*B*). Further, cell surface PAM fully reacts with hPg (Fig. 4*C*), a property of the N-terminal A-domain. We also show (Fig. 4, *D* and *E*) that each of the PAM variants expressed by the isogenic cells stimulates the activation of hPg by SK2b (GenBank: AMY98213.1), an SK subform that is coinherited with PAM-type Mprts (20). SK2b only enhances hPg activation when hPg is bound to PAM, which requires the N-terminal A-domain of PAM to be exposed to the extracellular solution to interact with hPg (20, 21). These results demonstrate that PAM and its variants bind in an equivalent and functional manner with hPg.

### Cleavage of peptides by subcellular fractions from SrtA-depleted AP53 cells

We expanded this study to further define the enzyme(s) responsible for the cleavage of PAM[T^355^Y]. Here, we used four isogenic AP53 GAS strains in various experiments: (1) AP53/*ΔsrtA*; (2) a double mutant deficient in both SrtA and SrtB (AP53/*ΔsrtA*/*ΔsrtB*); (3) a mutant deficient in SrtA and carrying the SrtA cleavage site mutation in PAM (AP53/*ΔsrtA*/*pam[T*^*355*^*Y])*, and (4), a targeted inactivation of an important membrane protease located at the Sec translocon channel, HtrA (GenBank: AMY98408.1), in a SrtA-deficient background (AP53/*ΔsrtA*/*ΔhtrA*).

**Figure 5:**
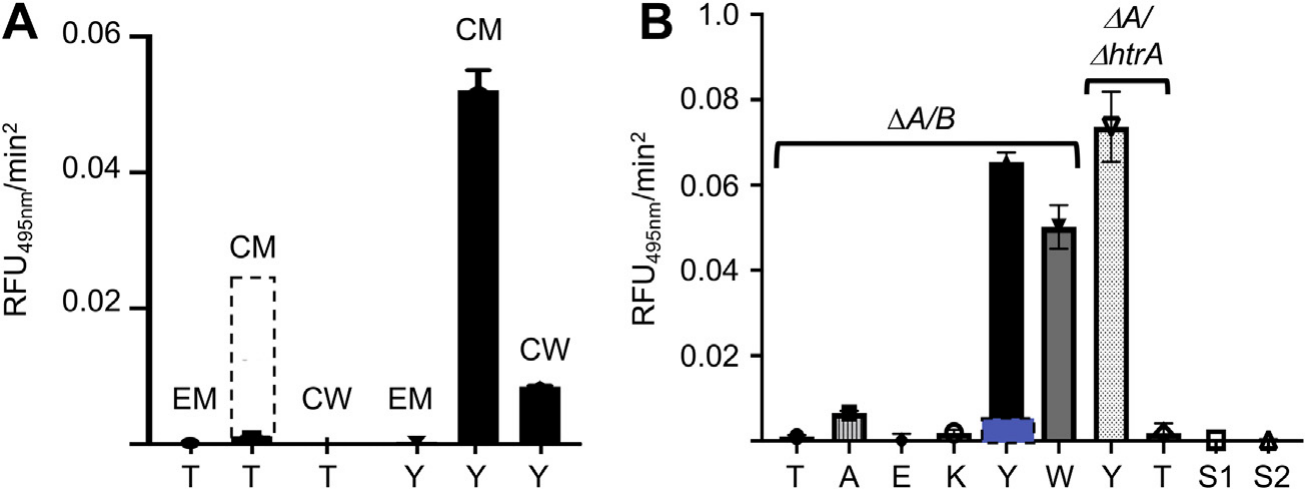
**Cleavage of synthetic peptides with AP53 subcellular fractions.***A*, cleavage of the synthetic peptides, Dabcyl-LPSTGEAA-Edans (T), and Dabcyl-LPSYGEAA-Edans (Y) with cell supernates (EM), cytoplasmic membranes (CMs), and cell wall (CW) fractions of WT-AP53 cells. The *dashed box* in the bar of the hydrolysis of Dabcyl-LPSTGEAA-Edans (T) is an experiment in which the reaction time with the same CM preparation was increased to 6 h in the presence of the SrtA stimulator, hydroxylamine. *B*, synthetic peptide cleavage by AP53/*ΔsrtA/ΔsrtB* CM fractions of the substrates: Dabcyl-LPSTGEAA-Edans (T), Dabcyl-LPSAGEAA-Edans (A), Dabcyl-LPSEGEAA-Edans (E), Dabcyl-LPSKGEAA-Edans (K), Dabcyl-LPSYGEAA-Edans (Y), and Dabcyl-LPSWGEAA-Edans (W) peptides, as well as two scrambled peptides, S1, Dabcyl-YLGPSEAA-Edans and S2, Dabcyl TLPGSEAA-Edans, are shown. The peptides (0.2 mg) were incubated for 3 h with AP53/*ΔsrtA/ΔsrtB*-derived CM fractions at room temperature. Cleavages of Dabcyl-LPSTGEAA-Edans and Dabcyl-LPSYGEAA-Edans peptides with CMs from the AP53/*ΔsrtA* and AP53*/ΔsrtA/ΔhtrA* (*ΔA/ΔhtrA*) isogenic cell lines. The *blue box* in the reaction of Dabcyl-LPSYGEAA-Edans with the AP53/*ΔsrtA/ΔsrtB* CMs under the same reaction conditions, except for the inclusion of the LPXTGase inhibitor, hydroxylamine. The peptides (0.2 mg) were incubated for 3 h with the indicated CM fractions at room temperature.

We first determined the subcellular fraction of the WT-AP53 cells that catalyzed cleavage of the WT-sequence, *viz*., Dabcyl-LPSTGEAA-Edans (T) and its variant, Dabcyl-LPSYGEAA-Edans (Y). From the results of Figure 5*A*, at dilutions and reaction times wherein the native peptide (LPSTGEAA) is slowly cleaved by all of the cell fractions, the same concentration of the variant peptide, Dabcyl-LPSYGEAA-Edans, is very rapidly cleaved by a component(s) in the CM. This comparative rate effect for Dabcyl-LPSTGEAA-Edans is seen by the dashed box in Figure 5*A*, where the reaction time of the peptide with the CM for AP53-WT cells was increased to 6 h in the presence of the SrtA-stimulatory nucleophile, hydroxylamine (15). The reaction was greatly stimulated under these conditions, as we expected since the strong nucleophile, hydroxylamine, is known to enhance serine and sulfhydryl proteases in which the mechanism of hydrolysis proceeds through acyl-intermediates (22).

Interestingly, in CMs from cells in which both the genes for both SrtA and SrtB are inactivated (AP53*/ΔsrtA/ΔsrtB*), as well as in cells in which the genes for *srtA* and *htrA* are inactivated (*AP53/ΔsrtA/ΔhtrA*), the variant (Y) peptide is still rapidly cleaved under conditions in which the peptide with the WT-sequence is cleaved very slowly (Fig. 5*B*). These cells were also grown with (1*S*,2*S*)-2-(((*S*)-1-((4-guanidinobutyl)amino)-4-methyl-1-oxopentan-2-yl)carbamoyl)cyclopropanecarboxylic acid (E64) in the medium, which is an inhibitor of the maturation of SpeB (23). This also eliminates SpeB as a candidate protease. Thus, the responsible protease(s) has a much higher specificity for aromatic residues at the fourth position of the SrtA cleavage site than for Thr at this same position in the WT-peptide. Further confirmation that the endopeptidase that catalyzed cleavage of the (Y) variant in the CM of cells with targeted deletions of the srtA and srtB genes is seen by large inhibition of its cleavage by hydroxylamine, an established inhibitor of LPXTGase (24).

### Distribution of PAM in subcellular fractions of AP53/***Δ****srtA* and AP53/***Δ****srtA*/*pam[T*^*355*^*Y]*

**Figure 6:**
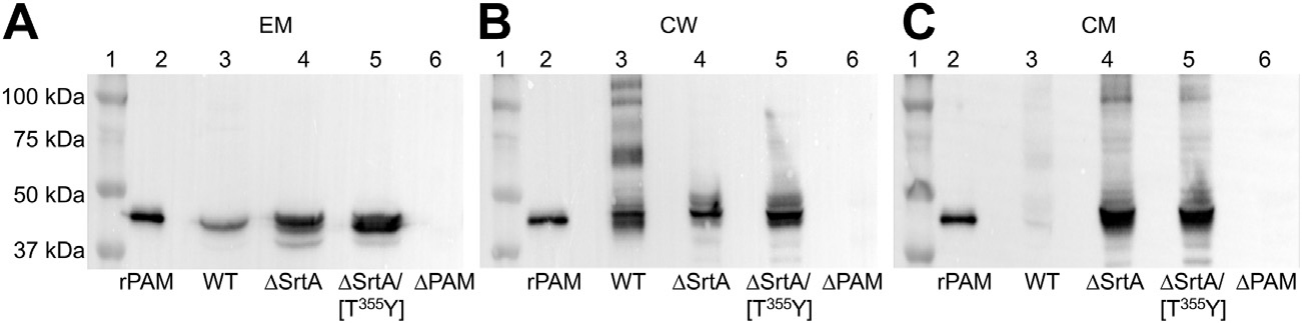
**Western blot analysis for PAM in AP53 cell fractions**. Isogenic AP53 strains were grown to mid-log phase and incubated with the indicated subcellular fractions that were isolated after PlyC digestion. The samples were applied to 10% tris-glycine SDS gels, transferred to PVDF membranes, and visualized with anti-PAM_short_. *A*, EM, cell supernate (40× concentrated). *B*, CW-cell wall. *C*, CM-cytoplasmic membrane. Lane 1, molecular weight standards; lane 2, rPAM; lane 3, WT-AP53; lane 4, AP53/*ΔsrtA*; lane 5, AP53/Δ*srtA/pam[T*^*355*^*Y]*; and lane 6, AP53/Δ*pam*. EM, extracellular membrane.

The data presented in Figure 6*A* demonstrate that AP53/*ΔsrtA/pam[T*^*355*^*Y]* cells secrete high levels of PAM[T^355^Y] into the EM (lane 5) that have been cleaved in a manner that yielded very similar molecular weights as rPAM (lane 2) (residues 1–351-His_6_, molecular weight = 40,941 Da). Additionally, PAM secreted by WT-AP53 cells (lane 3) and by AP53/*ΔsrtA* cells (lane 4) display molecular weights nearly identical to PAM secreted by WT-AP53 cells. The negative control cells, AP53/*Δpam*, did not show PAM secretion (lane 6), as anticipated.

The data of Figure 6*B* show that while PAM[T^355^Y] is cleaved from the CM in AP53/*ΔsrtA/pam[T*^*355*^*Y]* cells, it is not covalently linked to the CW since partially-digested CW peptidoglycan bands containing PAM[T^355^Y] are not observed (lane 5). This finding is similar to the CW distribution of PAM from AP53/*ΔsrtA* cells (lane 4). PAM from WT-AP53 cells, in which PAM is attached to incompletely digested peptidoglycan (lane 3), served as a positive control and the lack of PAM from AP53/*Δpam* cells (lane 6) provides the negative control.

The gels of Figure 6*C* show that WT-PAM is fully cleaved from the CM (lane 3) in WT-AP53 cells, while both WT-PAM (lane 4) and PAM[T^355^Y] (lane 5) are retained to a large extent in the CM in the absence of SrtA at molecular weights that suggest that the transmembrane domain is present, as required for embedding in the CM. Overall, the major conclusions of these experiments are that SrtA is not required for cleavage at or near the SrtA consensus site of PAM, but SrtA is necessary for transpeptidation to occur.

### Mass spectrometric analysis reveals the cleavage sites of WT and variant peptides by CM fractions

**Figure 7:**
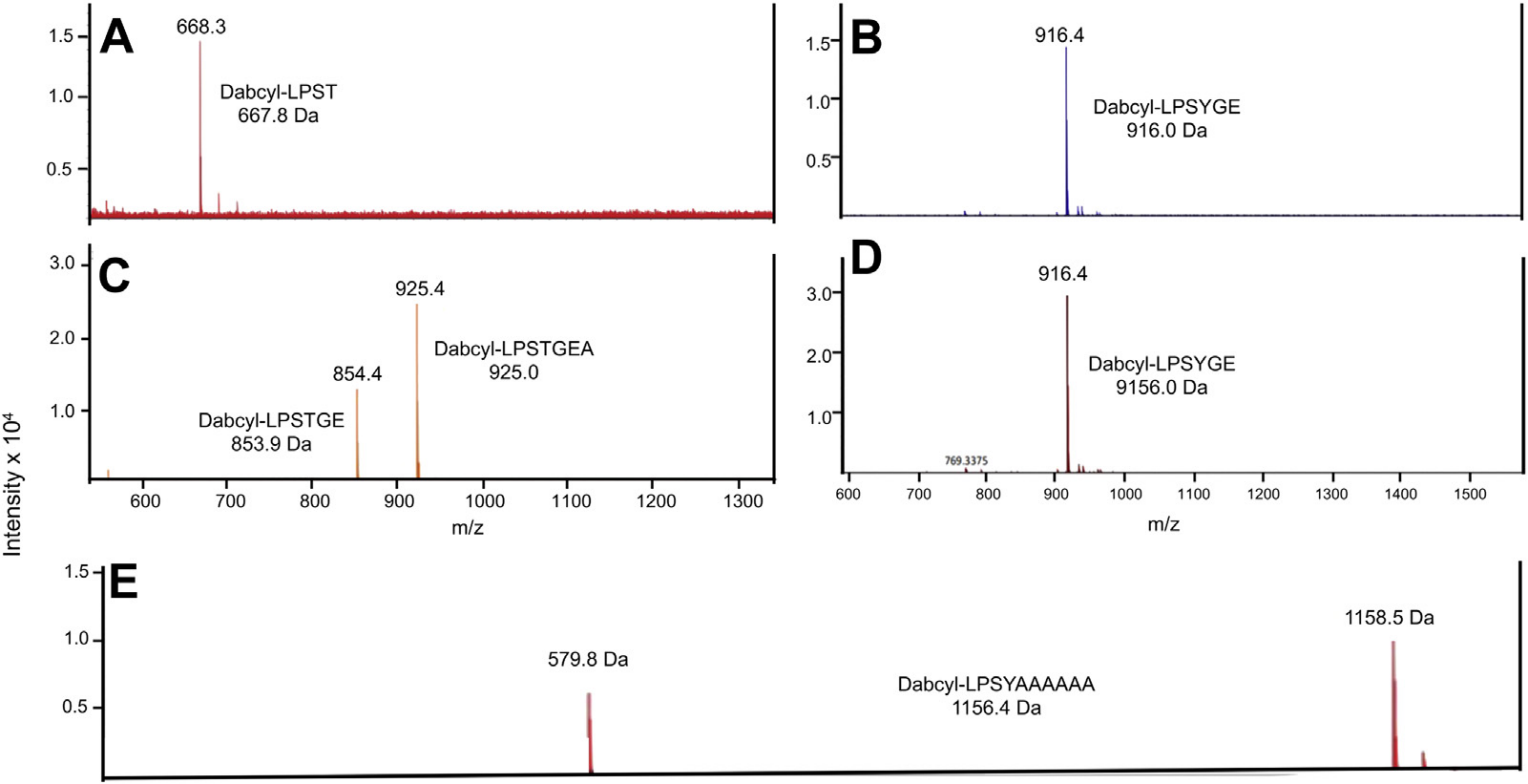
**Mass spectrometric analysis of the cleavage sites in synthetic peptides incubated withcytoplasmic membrane (CM)fractions**. Dabcyl-LPSTGEAA-Edans and Dabcyl-LPSTGEAA-Edans were incubated with AP53-WT or AP53/*ΔsrtA* CM fractions. *A*, Dabcyl-LPSTGEAA-Edans incubated with AP53-WT CM fractions shows a primary peak of mass 668.3 Da) corresponding to Dabcyl-LPST (calculated, 667.8). *B*, Dabcyl-LPSYGEAA-Edans incubated with the WT-AP53 CM fraction, showing a primary peak at 916.4 Da that corresponds to Dabcyl-LPSYGE (calculated, 916.0 Da). *C*, Dabcyl-LPSTGEAA-Edans incubated with CMs from AP53/Δ*srtA* cells shows two peaks, one at 854.4 Da corresponding to Dabcyl-LPSTGE (calculated, 853.9 Da) and a second peak at 925.4 Da corresponding to Dabcyl-LPSTGEA (calculated, 925.0 Da). *D*, Dabcyl-LPSYGEAA-Edans incubated with AP53/*ΔsrtA* CM fractions shows the primary peak at 916.4 Da corresponding to the Dabcyl-LPSYGE (calculated, 916 Da). *E*, Dabcyl-LPSYGEAA-Edans was incubated with the CM fraction from AP53-WT cells. The Dabcyl-containing product(s) possessed a parent mass of 1158.5 Da which corresponds to Dabcyl-LPSYAAAAAA (calculated m/z:1156.4 Da). The daughter mass at 578.9 Da is the one-half mass fragment of the parent peak.

To discover the exact location of the cleavage site in the peptides that are treated with CM fractions, we employed mass spectrophotometric analysis of the products of the cleaved peptides. Dabcyl-LPSTGEAA-Edans and Dabcyl-LPSYGEAA-Edans were incubated with CM fractions and the products that absorbed at the Dabcyl wavelength were subjected to mass analysis. When incubated with CM from WT-AP53, Dabcyl-LPSTGEAA-Edans showed a peak 668.3 Da (Fig. 7*A*), consistent with Dabcyl-LPST (calculated, 667.8 Da). This fragment is expected if SrtA catalyzed cleavage of this peptide. Upon incubation of Dabcyl-LPSYGEAA-Edans with WT-AP53 membranes, a peak 916.4 Da was observed (Fig. 7*B*), uniquely corresponding to Dabcyl-LPSYGE (calculated, 916.0 Da). This is characteristic of cleavage by LPXTGase, a nonribosomal membrane endopeptidase, discovered earlier (24). When Dabcyl-LPSTGEAA-Edans was incubated with CMs from AP53/[*ΔsrtA*] cells (Fig. 7*C*), two peaks were found, one at 854.4 Da which is the mass corresponding to Dabcyl-LPSTGE (calculated, 853.9 Da) and which is the main cleavage site for LPXTGase as well as an additional peak 925.4 Da, corresponding to Dabcyl-LPSTGEA (calculated 925.0 Da). Lastly, incubation of Dabcyl-LPSYGEAA-Edans with CMs from AP53/*ΔsrtA* cells show a primary peak at 916.4 Da (Fig. 7*D*), corresponding to Dabcyl-LPSYGE (calculated, 916 Da) which was previously determined to be the major cleavage site of LPXTGase (24).

While the nature of the products of the cleavage are shown in Figure 7, *A*–*D*, the percent yield at the incubation times of 24 h of each product is relevant. Dabcyl-LPSYGEAA-Edans reactions showed >98% cleavage by CMs from either WT (Fig. 7*B*) or AP53/*Δsrt*A (Fig. 7*D*) cells. However, Dabcyl-LPSTGEAA-Edans had a product yield of 50% with the CM from WT-AP53 cells (Fig. 7*A*) and only an 11% yield with the CM of AP53/*ΔsrtA* cells (Fig. 7*B*) at the times of the experiments, with the remainder being uncleaved peptide. This lower reaction yield of Dabcyl-LPSTGEAA-Edans by CMs from AP53/*ΔsrtA* cells confirms slower reaction rate of the WT-peptide to the remaining endopeptidase, LPXTGase.

In addition to these experiments, we examined the reaction product of Dabcyl-LPSYGEAA-Edans and hexa-alanine (AAAAAA) with CMs from WT-AP53 cells. The product displayed a mass peak at 1158.5 Da (in >60% yield), which corresponds to Dabcyl-LPSYAAAAAA (calculated m/z = 1156.4 Da). The half mass fragment of the parent peak generated in the mass spectrometer at 579.8 Da (in 40% yield) is also present.

The mass spectrometry data strongly suggest that the covalent attachment of PAM[T^355^Y] to the CW is through PAM[Y^355^] and strongly implicates SrtA as the participating endopeptidase.

### The variant PAM[T^355^Y] is functionally displayed on the AP53 cell surface

**Figure 8:**
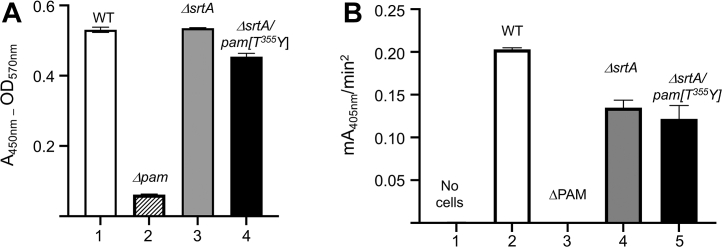
**Functional presence of PAM on whole cells.***A*, binding of anti-PAM to whole cells by ELISA. Mid-log phase isogenic AP53 cells were incubated with rabbit-anti PAM_short_ followed by goat-anti-rabbit IgG-HRP and developed with H_2_O_2_/TMB. The reaction was terminated with H_2_SO_4_ and the A_570nm_ (*light scatter*) was subtracted from the A_450nm_. Lane 1, WT-AP53; lane 2, AP53/*Δpam*; lane 3, AP53/Δ*srtA*; and lane 4, AP53/*ΔsrtA*/*pam[T*^*355*^*Y]*. *B*, stimulation of SK2b-catalyzed hPg activation by mid-log isogenic AP53 strains. Isogenic AP53 cells were placed in individual wells of a protein nonbinding 96-well microtiter plate, after which hPg was added, followed by S2251/SK2b. The hydrolysis of S2251 was continuously monitored at A_405nm_. The buffer was 10 mM Na-Hepes/150 mM NaCl, pH 7.4, at room temperature. The initial rates of activation are presented as *bar graphs*: lane 1, no cells; lane 2, WT-AP53; lane 3, AP53/Δ*pam*; lane 4, AP53/Δ*srtA*; and lane 5, AP53*/ΔsrtA/pam[T*^*355*^*Y]* cells. HRP, horseradish peroxidase; hPg, human plasminogen; TMB, tetramethylbenzidine.

We further show that PAMs from AP53/*ΔsrtA* and AP53/*ΔsrtA/pam[T*^*355*^*Y]* cells are displayed on the cell surface in the absence of SrtA. This was assessed by examining their interaction with anti-PAM_short_, indicating that all PAMs tested possessed a relatively intact N-terminus (Fig. 8*A*) with many, if not all, of the NH_2_-terminal 51 residues present in the product. The surface-exposed PAMs were also functional in stimulating hPg activation by SK2b (Fig. 8*B*). Whether extension from the CW, and/or from the CM, is the source of the surface-exposed PAM is not known at this stage, but an SrtA deficiency does not significantly affect the ability of CM-bound PAM to function in binding and stimulating the activation of hPg in whole AP53 cells (18).

**Figure 9:**
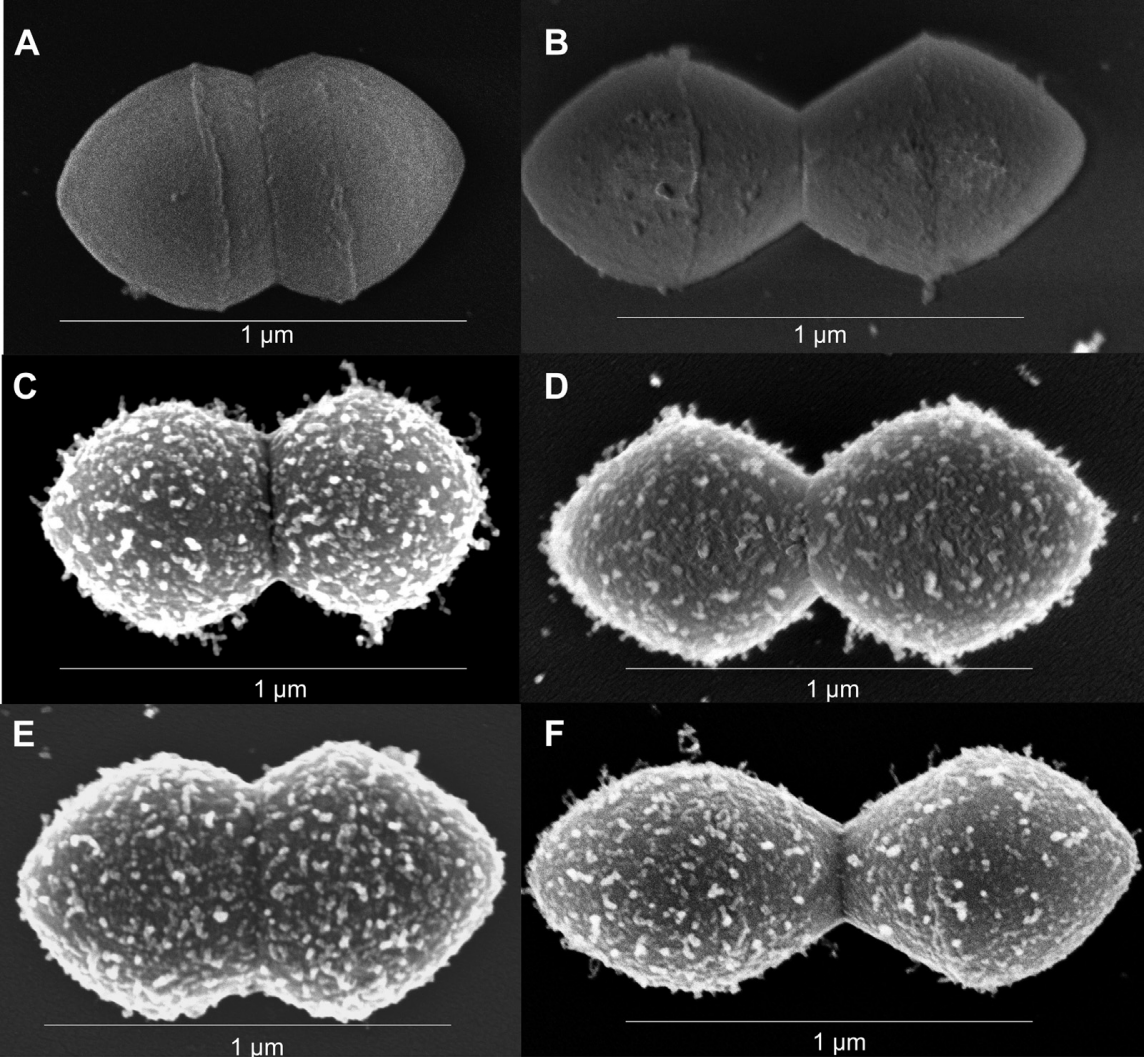
**SEM images of AP53 GAS cells**. Cells grown to mid-log phase and incubated with rabbit-anti PAM_short_ to determine the relative abundance of PAM on the surface of isogenic AP53 strains. *A*, WT-AP53, no antibody. *B*, AP53/Δ*pam* with rabbit-anti PAM_short_. *C*, WT-AP53 with rabbit-anti-PAM_short_. *D*, AP53*/pam[T*^*355*^*Y]* with rabbit-anti PAM_short_. *E*, AP53*/ΔsrtA/pam[T*^*355*^*Y]* with rabbit-anti PAM_short_. *F*, AP53/*ΔsrtA* with rabbit-anti PAM_short_.

The SEM results (Fig. 9) confirm all the findings of this article. The micrographs (Fig. 9, *A* and *B*) are negative controls showing that PAM is not detected on AP53 cells when anti-PAM is not present (Fig. 9*A*) or in cells with a targeted deletion of the *pam* gene (Fig. 9*B*). However, PAM is clearly visible on WT-AP53 cells (Fig. 9*C*) and in AP53/*pam[T*^*355*^*Y]* cells (Fig. 9*D*) treated with anti-PAM_short_. Finally, and importantly, WT-PAM (Fig. 9*E*) and PAM[T^355^Y] (Fig. 9*F*) are abundant on the cell surface when *srtA* gene is genetically deleted, likely extending from the CM to the surface of the cells.

## Discussion

Since Gram-positive bacterial strains do not have an outer membrane to stabilize surface-exposed proteins, specialized mechanisms of protein trafficking exist such that surface proteins are expressed in functional forms on the cell surface. The relatively extensive CW in these bacterial strains is of great utility in this regard. The study of surface proteome of bacterial pathogens is critical to their potential utility as vaccine candidates (25). In particular, vaccine candidates toward GAS have largely involved Mprt variants and other surface-exposed proteins as potential antigenic determinants for robust antibody-mediated responses (26, 27, 28). Considering our results, it is clear that further mechanistic studies are needed to better understand how GAS surface proteins, especially Mprt and PAM-protein variants, can be differentially localized and exposed to outer surfaces, and how variant LPXTG motifs may be a major mechanism by which GAS can quickly vary outer surface localization to increase antigenic variation.

To examine the surface proteins in GAS isolate, AP53, we mined the genomic sequence of this strain (17). A total of 28 candidate lipoproteins with a defined lipobox were found. These proteins are imbedded in the CM by posttranslational N-terminal anchoring that enables surface display of their C-terminal regions by extension from the CM, through the CW and to the outer capsule (29). Additionally, several moonlighting proteins, especially cytosolic enzymes involved in glycolysis, for example, enolase and GAPDH, despite not employing signal sequences, are also found on the surface of AP53 cells (2, 3, 30). Their cell surface trafficking pathways from the cytosol are largely unknown. We also identified ∼17 proteins that contain the SrtA signal sequence, one (PrtS/CspC) with an Ala at the fourth position of the SrtA cleavage motif and the remaining 16 proteins possessing Thr at this cleavage site. Biologically relevant cleavages of the Tyr or Trp mutants at the SrtA cleavage site were heretofore unknown.

We show that cells containing PAM[T^355^Y] (Tyr substituted for Thr at the fourth position) of the SrtA cleavage motif display the same mid-log phase subcellular distribution as cells containing WT-PAM, and this PAM variant is covalently attached to the CW, through Tyr^355^. Both WT-PAM and PAM[T^355^Y] are observed in the EM at molecular weights that suggest that these proteins have been similarly cleaved from the CM. We presume that when Tyr is substituted for Thr fourth position at the PAM cleavage site is poorly cleaved by SrtA *in vitro* under the chosen conditions, or with isolated CMs, cleavage by SrtA in cells occurs at a sufficient rate to place this PAM mutant on the CW, especially given the time that the cells have to reach the mid-log growth stage and with the likelihood of proximity effects, since the components are present together at or near the Sec translocon channel. Importantly, the SrtA-bound PAM[T^355^Y] is transpeptidated to the peptidoglycan to approximately the same extent as the natural PAM[T^355^].

The CM-spanning Sec translocon, consisting of the SecYEG protein triheteromer, as well as ancillary proteins, including SecA, transports unfolded proteins containing a signal sequence, for example, PAM, from the cytoplasm through the CM, where other processing proteins, particularly SrtA, can function to deliver the protein to its final destination (31). Sec translocons are highly concentrated in a CM microdomain organelle termed the Ex Portal (32). If these proteins are misfolded, they are degraded by HtrA. We show that when SrtA is inactivated, PAM is delayed in the CM where it is cleaved by another specific protease, within the SrtA site, but is not transpeptidated to the CW. In this case, PAM is found both noncovalently bound within the CM, where it is still functional regarding hPg binding and activation, and free in the EM. We hypothesized that PAM delayed in the CM nonetheless is able to span the CW to present its N-terminus on the cell surface, where it is able to interact with hPg through the exposed A-domain (18). Also, PAM that is cleaved within the LPXTG cleavage site, but not transpeptidated to the peptidoglycan, can also expose its A-domain for the same purpose.

Since an SrtA inactivation affects many other proteins of GAS, we attempted another manner of inhibiting SrtA cleavage, specifically using PAM, that would support our hypothesis. PAM is cleaved *via* SrtA between the fourth (Thr) and fifth (Gly) position (at ^↓^) of the LPST^↓^GEAA consensus SrtA sequence, immediately upstream of its TMD near the C-terminus. Thus, we sought a peptide sequence that would be highly attenuated toward SrtA cleavage, specifically in PAM. We first investigated the activation rates catalyzed by SrtA in a series of LPSXGEAA peptides *in vitro* and found that LPSTGEAA is the most rapidly cleaved peptide, as expected. The next best peptide sequence for cleavage contains Lys in place of Thr at the fourth position of the cleavage site, followed by an Ala at this position, a known biologically relevant residue in SrtA-sensitive proteins. *In vitro*, other peptides with substitutions at the fourth position are cleaved at slower rates, with LPSYGEAA and LPSWGEAA as the most unfavorable substrates for rSrtA. Thus, we generated an AP53 cell line with a targeted replacement of WT-PAM by PAM[T^355^Y]. Surprisingly, we found that *in vivo*, the variant PAM was distributed in AP53 cellular fractions similarly to WT-PAM, in that PAM[T^355^Y] was transpeptidated to the peptidoglycan to approximately the same extent as WT-PAM under or conditions of cell growth. This shows that the low rate of activity of the LPSYGEAA peptide toward SrtA *in vitro* was nonetheless sufficient for the SrtA reactions to occur *in vivo*.

A different picture emerges when isolated CMs from WT-AP53 cells are compared to AP53/*ΔsrtA/ΔsrtB* cells in their ability to cleave the synthetic peptides, LPSTGEAA, LPSYGEAA, and LPSWGEAA. Here, an endopeptidase found in the CM cleaves within the SrtA cleavage site but apparently does not transpeptidate the PAMs to the peptidoglycan. An explanation for these finding lies with the presence of a unique membrane endopeptidase, the nonribosomally synthesized LPXTGase, or a similar unknown endoprotease, which cleaves within the SrtA cleavage motif at position-3 (Ser^3^) and position-5 (Glu^5^) of the WT-LPSTG sequence (Ser^354^, Glu^357^ of PAM) (33), as well as at P3’ (Ala^7^; Ala^358^ of the WT-LPSTGEAA sequence). LPXTGase is an unusual enzyme in that it is very hydrophobic, heavily glycosylated, and contains unconventional amino acids, as well as D-alanine, in its amino acid composition (24). It is believed that this enzyme is nonribosomally produced and is assembled in part by the enzymes used to construct the CW (33). According to our results herein, we conclude that this enzyme prefers aromatic residues at the P1 position of the SrtA cleavage sequence and has lower activity when the natural Thr is present at this location, a favorable situation for GAS viability.

In considering other proteases that may function in place of SrtA and an enzyme like LPXTGase, obvious choices are CM-bound HtrA (GenBank: AMY94808.1) and SpeB (GenBank: AMY98254.1), although SpeB is a secreted protein and is unlikely to function in this regard. The *htrA* gene encodes the serine protease, HtrA, and has a noncleavable signal peptide, as well as a TMD near the amino terminus of the protein (34). Thus, this protease is anchored in the CM by its amino terminus, where it is located at the Sec translocon domains of the Ex Portal of the CM (32).

The role of HtrA is to catalyze degradation of misfolded proteins secreted through the Sec channel and to act as a protease to assist the maturation process of some secreted proteins (35). HtrA also serves as a stress-response protein and has chaperone-like properties, which may help the organism in avoiding oxidative stress (36). Because of these properties, we constructed a targeted gene deletion of *htrA* in a SrtA-deleted background (AP53/Δ*srtA/ΔhtrA*) and grew these cells to mid-log phase. The SpeB maturation inhibitor (E64) (37) was also present in the growth media, thus also inhibiting SpeB. Isolation of the CM from these cells and incubation of the peptide, Dabcyl-LPSTGEAA-Edans, with this CM fraction still led to very little endopeptidase activity. On the other hand, treatment of Dabcyl-LPSYGEAA-Edans with these CMs still display very high activity, thus eliminating HtrA and SpeB, along with SrtA or SrtB, as candidate CM proteases that cleaved Dabcyl-LPSYGEAA-Edans. Thus, we are left with the inescapable conclusion that the endopeptidase, LPXTGase is the enzyme in the CM that displays low activity to the WT-peptide, thus allowing SrtA to function in the presence of LPXTGase, and very high activity toward the mutant peptide, Dabcyl-LPSYGEAA-Edans, where SrtA exhibits a lower level of activity.

The major conclusions reached in this study are verified by the SEM images of the isogenic cell lines. WT-AP53 cells react with anti-PAM_short_, showing an abundance of PAM on the cell surface, as is also the case with AP53/*ΔsrtA* cells. This confirms that through its elongated structure, PAM exposes its N-terminal hPg-binding domain at the cell surface while its C-terminus is trapped in the CM through its TMD. PAM is also covalently linked through its SrtA consensus site to CW peptidoglycan. The SEM scans similarly show that PAM[T^355^Y] also extends through the CW, exposing its N-terminus to the EM, with or without SrtA. Thus, while SrtA is necessary to permanently link PAM to the peptidoglycan (38), this step is not required to expose PAM on the cell surface to bind and activate hPg.

In conclusion, the results of this article are consistent with a hypothesis that the nonribosomal primordial endopeptidase, LPXTGase, or another as yet undiscovered nonribosomal endoprotease, was present in CMs of ancient species of bacteria and formed at the time of initial bacterial CW synthesis. SrtA evolved when it was advantageous for bacteria to have stable cell surfaces to interact with potential host cells. In the case of Gram-positive bacteria, covalent attachment of proteins to their large CWs was an efficient manner of providing these stable cell surfaces. For maximum efficiency of CW attachment, Sec channels with associated Srts evolved and organized within a membrane microdomain, the Ex Portal. This not only developed and grouped efficient multiple Sec channels for cleavages at specific sites but also for protected potential substrates from other CM proteases not present in the Sec channel, for example, the highly active LPXTGase.

## Experimental procedures

### GAS strain

GAS isolate, AP53 (emm53), was obtained from Dr G. Lindahl, Lund, SE.

### Proteins

#### Cloning and expression of recombinant SrtA and SrtB.

SrtA (GenBank: AMY97447.1), residues 82 to 249 ([V^82^]SrtA), without its predicted N-terminal signal peptide nor its TMD and peripheral membrane region, was cloned from GAS-AP53 genomic DNA (gDNA) (GenBank: CP013672.1) using a 21 bp forward primer beginning at the first nucleotide encoding V^82^ and a 30 bp reverse primer beginning at the stop codon for *srtA*. The reverse primer included introduction of a new *BamH1* site after the *srtA* stop codon. After digestion with *BamH1*, the PCR products were ligated into *PshA1/BamH1*-digested pET42a (EMD Biosciences), yielding the *srtA* cDNA with nucleotides encoding an additional N-terminal 276-amino acids from pET42a. The entire expression cassette contained, sequentially, a GST tag, a 15 residue S-tag (39), a (His)_6_ tag for purification, a Factor Xa (FXa) cleavage site (IEGR^↓^), followed by [V^82^]SrtA, which is the final product after purification and FXa cleavage.

The same procedure was used to prepare the plasmid for *srtB* expression (GenBank: AMY96673.1), lacking the N-terminal 36-residue signal peptide and its TMD, and providing residues 37 to 241 ([H^37^]SrtB).

The plasmids were expressed in *E. coli* BL21/DE3 cells as we have described previously in detail for proteins such as PAM, PAM fragments, and SK (20, 40, 41). After purification by Ni^+^-charged His-trap resin (EMD Biosciences), elution with imidazole and then cleavage of the FXa-sensitive site (at R), the final purified SrtA proteins consisted of [V^82^]SrtA or [H^37^]SrtB, without non-native amino acids (20).

All PCR reactions were carried out with Phusion Hot Start High-Fidelity DNA Polymerase (New England Biolabs).

hPg binding Group A streptococcal Mprt (PAM_AP53_) was expressed in *E. coli* and purified as described (41). The final recombinant PAM product consisted of residues 1 to 351 + an additional COOH-terminal (His)_6_.

*Streptokinase cluster 2b (SK2b)* was also expressed in *E. coli* as previously described (20).

hPg was expressed in *Drosophila Schneider S2* cells and purified by Sepharose-lysine affinity chromatography, as described (42).

#### Antibodies

Polyclonal antibodies against PAM were generated in rabbits by standard procedures. Similarly, a rabbit polyclonal antibody was generated against a form of PAM, containing only the intact N-terminal 132 amino acids (PAM_short_, residues 1–132, lacking the 41-residue signal peptide; Fig. 1*B*). This PAM fragment contained the full hypervariable domain (HVD) (+1), the full hPg binding A-domain, and the B domain. Additionally, anti-PAM_short_ showed no reactivity with VEK50 (Fig. 1*C*), a form of PAM (residues 55–104) that contains the C-terminus of the HVD, the full hPg binding A-domain, and the N-terminus of the B-domain. This implies that anti-PAM_short_ requires the N-terminal region of the HVD for reactivity.

### Isogenic AP53 strains

The generation of the isogenic AP53 strain with a targeted deletion of the entire *srtA* gene has been described earlier (18, 43).

Allelic replacement by the *cat* gene of the 726 bp *srtB* gene was similarly conducted. The targeting plasmid contained the 660 bp *cat* gene, flanked by 468 bp upstream of the ATG signal for *srtB* and 378 bp downstream of its TAA stop codon. During this construction, 5′-*NotI* and 3′-*XhoI* restriction sites were also cloned into this DNA segment using PCR primers. These sites were employed for insertion of the targeting plasmid into the same sites of the temperature-sensitive plasmid pHY304 (from M. J. Walker), which also contained the downstream erythromycin resistance (*erm*) gene. Integration of *cat* into the *srtB* chromosomal locus was accomplished by single crossover (SCO) at 30 °C and double crossover (DCO) at 37 °C, which also eliminated the *erm* gene. Screening of confirmed SCO colonies was conducted by gain of erythromycin resistance. DCO colonies obtained from the confirmed SCO colonies were screened by loss of erythromycin resistance after elimination of the *erm* gene during DCO. DCO colonies were further tested by resistance to chloramphenicol. The substitution of *cat* for *srtB* was additionally confirmed by PCR of the genomic DNA with *cat* and *srtB* specific primers.

The AP53/*ΔsrtA/ΔsrtB* double deletions were constructed as above, with the targeting plasmid containing *ΔsrtB* incorporated into the AP53/*ΔsrtA* cells by DCO as above.

The same general procedures were used for generation of *pam[T*^*355*^*A]* and *pam[T*^*355*^*Y]* in AP53 cells or in AP53/*ΔsrtA* cells. The DCO colonies were screened using primers specific for the LPSAG and LPSYG motifs and Sanger sequencing of the *pam* genes.

Replacement of the entire coding sequence for *htrA* (GenBank: AMY94808.1) in a *srtA*-deficient background (AP53/*ΔsrtA/ΔhtrA*) was similarly constructed. The targeting plasmid contained 593 bp upstream of the ATG signal for *htrA* and 349 bp downstream of its TAA stop codon. An A^7^T single nucleotide change was placed in the 349 bp 3′-flank to generate a new restriction site *HindIII* for genome mapping. During this construction, 5′-*EcoRI* and 3′-*XhoI* restriction sites were also cloned into this targeting DNA segment using PCR primers and employed for insertion into pHY304 as above. Integration of the targeting plasmid into the *htrA* chromosomal locus in AP53/*ΔsrtA* cells was then accomplished by SCO at 30 °C and DCO at 37 °C. Screening of confirmed SCO colonies for DCO lines was conducted by loss of erythromycin resistance after the DCO. DCO colonies were further PCR-genotyped for elimination of the entire *htrA* gene sequence using internal *htrA* primers. The substitution of a new *HindIII* site in the DCO insert was further confirmed by *HindIII* digestion of the PCR product of the gDNA with specific 5′-external and 3′-external primers.

### Genotyping

gDNAs were prepared from single colonies of AP53 lines selected from sheep blood agar plates. The colonies were treated with lysozyme/proteinase K and suspended in 100 mM Tris/5 mM EDTA/0.2% SDS/200 mM NaCl, pH 8.5. gDNAs were extracted with phenol/chloroform/isoamyl alcohol (25/24/1), precipitated with isopropanol, and washed with 70% ethanol in nuclease-free H_2_O.

PCR was employed with gDNA from the various strains to determine whether the desired gene alterations were present in the genomes. To detect the *srtA* gene, 24 bp internal primers that consisted of a forward primer *(srtA-F)* beginning at nucleotide 233 of *srtA* and a reverse primer *(srtA-R)* beginning at nucleotide 492 of *srtA* were used. The *srtA* gene showed a 260 bp amplicon with these primers.

For detection of the *cat* gene, 20 bp and 21 bp primers were employed beginning at nucleotide 241 for the forward primer (*cat-F*) of the *cat* DNA and at nucleotide 460 for the reverse primer (*cat-R*) of the *cat* cDNA. This provided a 220 bp amplicon when the *cat* gene was present.

To detect the *srtB* gene, a 19 bp forward primer (*srtB-F*) beginning at nucleotide 82 and a 20 bp reverse primer (*srtB-R*) beginning at nucleotide 317 was used to yield an amplicon of 236 bp.

For detection of *WT*-*pam*, an 18 bp forward primer (*pam-F*) beginning at nucleotide 81 of the *pam* gene and a 20 bp reverse primer (*pam-R*) which began at 439 bp of the *pam* gene yielded a 359 bp amplicon when *pam* was present.

For detection of *pam[T*^*355*^*A]*, a 22 bp forward primer (*pam[T*^*355*^*A]-F)* beginning at nucleotide 1081 and a 23 bp reverse primer (*pam[T*^*355*^*A]-R)* beginning at nucleotide 1387 yielded an amplicon of 307 bp. For further screening, an *Afe1* restriction site was placed in the mutagenesis primer which digested the 307 amplicon into 109 bp and 198 bp fragments when the PAM[T^*3*55^*A*] mutation was present in either WT-AP53 cells or in AP53/*ΔsrtA* cells.

Similarly, to detect the *pam[T*^*355*^*Y]* mutation in WT-AP53 cells (AP53/*pam[T*^*355*^*Y*]) or AP53/*ΔsrtA* cells (AP53/*ΔsrtA/pam[T*^*355*^*Y*]), the same primers were used as for *pam[T*^*355*^*A]*, except that a new *NdeI* restriction site was present in the 307 bp amplicon. Digestion of the amplicon with *NdeI* provided 109 bp and 198 bp fragments when the *pam[T*^*355*^*Y]* mutation was present in either AP53 cells or AP53/*ΔsrtA* cells.

Also, PCR of the gDNAs using an external 5′-primer upstream of the *srtA* or *pam* gene with *cat-R* and PCR of gDNAs with an external reverse primer downstream of the *srtA* or *pam* genes with *cat-F* provided correct amplicons in all cases, showing that *cat* and *pam genes* were appropriately targeted in each of the strains.

To detect the *htrA* gene, 21 bp and 22 bp internal primers that consisted of a forward primer beginning at nucleotide 122 of *htrA* and a reverse primer beginning at nucleotide 409 of *htrA*, respectively, were used. The presence of the *htrA* gene provided a 288 bp amplicon. To detect the *htrA* deletion, a 21 bp 5′-external forward primer 24 bp upstream of the ATG of *htrA* and 24 bp 3′-external reverse primer downstream of the TAA of *htrA* provided a 1168 bp amplicon in the deletion allele, as compared to a 2392 bp amplicon when *htrA* is present.

### Preparation of the CW, CM, and cytoplasm from AP53 cells

Overnight cultures of the different strains of AP53 cells were grown to mid-log phase (OD_600nm_ = 0.55–0.6) in Todd Hewitt medium supplemented with 1% yeast extract at 37 °C/5% CO_2_. The cultures (40 ml) were pelleted in a table-top centrifuge, and the supernates (200 μl) concentrated 40× for Western analysis with rabbit-anti-PAM_short_.

The resulting pellets were washed with PBS, then resuspended in 1 ml PBS-30% raffinose/20 μg bacteriophage Ply-C (44), and incubated for 1 h at room temperature. The resulting samples were then centrifuged for 10 min to provide the CW in the supernatants and the spheroplasts in the pellets. The CW fractions were removed and the spheroplasts were lysed by the addition of 1 ml sterile H_2_O, then vortexed to induce further lysis, and finally digested with 30 μl DNase I (2 units/μl) at 37 °C for 30 min. Next, the samples were centrifuged at 165,000*g* for 3 h at 4 °C. The cytoplasm was removed as the supernatant and the CM pellet was then resuspended in 1 ml sterile H_2_O. The CW and CM samples were employed for Western blotting and fluorescent peptide cleavage assays.

### Cleavage of fluorescence-labeled peptides by recombinant (r)-Srts

Dabcyl (4-((4-(dimethylamino)phenyl)azo)benzoic acid, succinimidyl ester)-LPSXGEAA-(5-[(2-aminoethyl)amino]naphthalene-1-sulfonic acid) (Edans) peptides and two scrambled versions of peptides of containing Thr^355^ (Dabcyl-TLPGSEAA-Edans) and Tyr^355^ (Dabcyl-YLGPSEAA-Edans) were synthesized by GenScript. Six peptide sequences were selected for study, *viz*., Dabcyl-LPSTGEAA-Edans, Dabcyl-LPSAGEAA-Edans, Dabcyl-LPSEGEAA-Edans, Dabcyl-LPSKGEAA-Edans, Dabcyl-LPSYGEAA-Edans, and Dabcyl-LPSWGEAA-Edans. The fluorescent peptides (0.2 mg) were dissolved in 1 ml 50 mM NaCl/50 mM Tris–HCl/0.2 M NH_2_OH, pH 7.4. An aliquot of 100 μl of the peptide solution was transferred to replicate wells of a 96-well black fluorescence plate, and 100 μl of 0.4 μM SrtA or SrtB was added to the individual wells. Wells which contained only buffer and peptides were used as controls. Fluorescence was measured at excitation and emission wavelengths of 350 nm and 495 nm, respectively, every 30 s for 3 h at 37 °C. The change in fluorescence was calculated and the slope of the line by linear regression was determined using GraphPad Prism 9.

The same procedure was employed for cleavage of these peptides by the EM, CW, and CM fractions of the AP53 isogenic cells.

### Mass spectrometry

Dabcyl-LPSTGEAA-Edans and Dabcyl-LPSYGEAA-Edans peptides were dissolved in 50 mM Tris–HCl/50 mM NaCl, pH 7.4. The peptide solutions were incubated with 5 μl AP53-WT or AP53/[ΔSrtA] CM fractions for 24 h, then passed over a C18 desalting column equilibrated with 5% acetonitrile/0.5% TFA, and finally eluted with 70% acetonitrile in H_2_O. The samples were applied to a Bruker Impact II UPLC system. Ultra performance liquid chromatography fractions were separated by elution times through the column with the major peaks eluting between 9 to 9.5 min for all four samples. These fractions were tested using UV-Visible spectroscopy to determine if they absorbed light at the wavelength (495 mm) for Dabcyl. Fractions that were shown to absorb at 495 nm were processed by mass spectrometry on a Bruker Impact II Very High-Resolution Quadrapole Time-of-Flight Mass Spectrometer. The masses of the fractions were determined. A background of 70% acetonitrile in H_2_O was taken and peaks from the solvent were subtracted from the mass spectrometry results to remove background noise.

### Transpeptidation assay

Hexa-alanine was prepared using solid phase peptide synthesis starting with Fmoc-Ala-Wang resin. The Fmoc was removed with piperidine, and Fmoc-Ala was coupled to the deprotected resin-bound Ala in the usual fashion. These steps were repeated for a total of five rounds. After each deprotection, the resin was checked with ninhydrin for completeness of deprotection. When the synthesis was completed, the peptide was cleaved from a small sample of the resin. A single peak was obtained by HPLC. The mass of the sample was found to be 445.2 Da (calculated, 444.5 Da), corresponding to (Ala)_6_.

Dabcyl-LPSYGEAA-Edans, dissolved in 50 mM Tris–HCl/50 mM NaCl, pH 7.4, was added to deprotected (Ala)_6_-Wang resin. Following this, 40 μl of the CM fraction from WT-AP53 cells was added. After 24 h, the resin was centrifuged and the supernatant was removed. Next, the resin was washed 3× with 20 μl TFA. In order to cleave the product from the resin, the sample was incubated for 3 h in 250 μl TFA. The resin was filtered and the TFA product was collected. The cleaved peptide fraction was precipitated with 750 μl diethyl ether. After removal of the diethyl ether, the sample was dried overnight, then dissolved in 100 μl H_2_O, and analyzed by mass spectrometry as above.

### Binding of hPg to AP53 cells by flow cytometric analysis

Mid-log phase AP53 cells were grown as above. All succeeding steps were accomplished after suitable washings to remove unbound materials. Bacteria cells were pelleted and resuspended in PBS/1% bovine serum albumin blocking solution for 30 min. After this, ∼3 × 10^8^ cells were incubated with hPg (200 nM) for 1 h at 25 °C and then incubated for 30 min with monoclonal mouse-anti-hPg IgG (ERL). The AP53-bound hPg was detected by incubation with Alexa Fluor 488-donkey-anti-mouse IgG (Invitrogen) in the dark for 30 min. Finally, the cells were fixed in PBS/1% paraformaldehyde. Fluorescence data were acquired, using the BD FACSAria III (BD Biosciences) by gating on fluorescence (FITC-A) and side-scatter, with scales set to logarithmic amplification. The cells in suspension were analyzed at a flow rate of 10 μl/min and 10,000 events per analysis. Histograms were analyzed using FCS Express Version 4 software (*De Novo* Software). The median fluorescence intensity, which measures the ability of each isogenic strain to bind hPg, was plotted against the respective strain using GraphPad Prism 9.

The binding of rabbit-anti PAM or rabbit-anti-PAM_short_ to the isogenic AP53 cells was carried out in a similar way as described for hPg binding, except that the cells were incubated with rabbit-anti-rPAM polyclonal antibody for 30 min and next incubated with Alexa Fluor 488-chicken-anti-rabbit IgG (Invitrogen) in the dark for 30 min.

### Binding of anti-PAM to AP53 cells by whole cell ELISA

Mid-log phase AP53 cells were prepared as above. After 30 min incubation in PBS/1% BSA, ∼3 × 10^8^ cells were transferred to microcentrifuge tubes and incubated with rabbit-anti PAM_short_ for 30 min. The cells were centrifuged, followed by a 30 min incubation with goat-anti-rabbit IgG-horseradish peroxidase. Next, the cells were suspended in PBS, and the A_600nm_ was adjusted to ∼0.16. Cell suspensions were diluted 5× and 50 μl of the suspensions were added in triplicate to individual wells of a 96-well microtiter plate. Color development was initiated by the addition of 100 μl horseradish peroxidase substrates, H_2_O_2_ and 3,3′,5,5′-tetramethylbenzidine. The reaction was allowed to proceed for 10 min at room temperature, after which it was terminated by the addition 50 μl 2N H_2_SO_4._ The A_450nm_ and A_570nm_ were determined. The A_570nm_ was subtracted from the A_450nm_ to correct for light scattering from bacterial cells. The data were plotted using GraphPad Prism software, version 9.

### Activation of hPg by AP53 whole cells

The procedure for the hPg continuous activation assay by SK2b has been described using mid-log phase AP53 cells. The bacteria were incubated in PBS/1% BSA for 30 min, and ∼2 × 10^8^ cells were added to individual wells of a 96-well microtiter plate. Blank wells contained PBS/1% BSA with no added cells. Next, hPg (200 nM, final concentration) was added to all wells, followed by 15 min incubation. At this point, the chromogenic substrate, H-D-Val-L-Leu-L-Lys-p-nitroanilide (S2251), and SK2b in 10 mM Na-Hepes/150 mM NaCl, pH 7.4, were added together to final concentrations of 0.25 mM and 5 nM, respectively. The hPm-catalyzed release of p-nitroaniline from S2251 was continuously monitored by A_405nm_ for up to 120 min. Initial velocities of activation were calculated as the slope of the linear region of plots of A_405nm_ *versus* t^2^ (45). The data were analyzed using GraphPad Prism Version 9.

### Scanning electron microscopy imaging of specific antibodies bound to AP53 GAS cells

Cultures of isogenic AP53 GAS cells were grown at 37 °C/5% CO_2_ in Todd Hewitt medium supplemented with 1% yeast extract until the mid-log phase was reached (A_600nm_ = 0.55–0.60). All strains grew at approximately the same rates. The cultures (40 ml) were then pelleted using a routine tabletop centrifuge, washed with PBS, and then incubated for 1 h with rabbit-anti-PAM, rabbit-anti-PAM_short_, or PBS. Subsequent to this step, the cells were then again pelleted, and the cell pellets were placed in 2% glutaraldehyde/0.1 M sodium cacodylate in PBS. A volume of 2 μl was spotted onto glass microscope slides coated with 0.01% poly-L-lysine in PBS for cross-linking to occur. The slides were then rinsed with PBS and then incubated for 1 h in 1% OsO_4_ in PBS and finally rinsed with PBS. The samples were then dehydrated stepwise using 50%, 70%, 80%, 95%, and 100% ethanol for 10 min. Ethanol was then removed, and samples were submerged in liquid CO_2_ and allowed to reach the critical point to dry. The slides were then attached to SEM stubs and sputter coated with gold to 3 nm thickness. Samples were imaged at 800,00× using the FESEM-Magellan 400 (FEI).

## Data availability

All data are supplied in this article.

## Conflicts of interest

The authors declare that they have no conflicts of interest with the contents of this article.
